# Early surgical reconstruction versus rehabilitation with elective delayed reconstruction for patients with anterior cruciate ligament rupture: COMPARE randomised controlled trial

**DOI:** 10.1136/bmj.n375

**Published:** 2021-03-09

**Authors:** Max Reijman, Vincent Eggerding, Eline van Es, Ewoud van Arkel, Igor van den Brand, Joost van Linge, Jacco Zijl, Erwin Waarsing, Sita Bierma-Zeinstra, Duncan Meuffels

**Affiliations:** 1 Department of Orthopaedics and Sports Medicine, Room NC-424, Erasmus MC University Medical Centre, PO Box 2040, 3000 CA Rotterdam, Netherlands; 2 Department of Orthopaedics, Haaglanden Medical Centre, The Hague, Netherlands; 3 Department of Orthopaedics, Elisabeth Tweesteden Hospital, Tilburg, Netherlands; 4 Department of Orthopaedics, Reinier de Graaf Gasthuis, Delft, Netherlands; 5 Department of Orthopaedics, St Antonius Hospital, Nieuwegein, Netherlands; 6 Department of General Practice, Erasmus MC University Medical Centre, Rotterdam, Netherlands

## Abstract

**Objective:**

To assess whether a clinically relevant difference exists in patients’ perceptions of symptoms, knee function, and ability to participate in sports over a period of two years after rupture of the anterior cruciate ligament (ACL) between two commonly used treatment regimens.

**Design:**

Open labelled, multicentre, parallel randomised controlled trial (COMPARE).

**Setting:**

Six hospitals in the Netherlands, between May 2011 and April 2016.

**Participants:**

Patients aged 18 to 65 with an acute rupture of the ACL, recruited from six hospitals. Patients were evaluated at three, six, nine, 12, and 24 months.

**Interventions:**

85 patients were randomised to early ACL reconstruction and 82 to rehabilitation followed by optional delayed ACL reconstruction after a three month period (primary non-operative treatment).

**Main outcomes:**

Patients’ perceptions of symptoms, knee function, and ability to participate in sporting activities were assessed with the International Knee Documentation Committee score (optimum score 100) at each time point over 24 months.

**Results:**

Between May 2011 and April 2016, 167 patients were enrolled in the study and randomised to one of two treatments (mean age 31.3; 67 (40.%) women), and 163 (98%) completed the trial. In the rehabilitation and optional delayed ACL reconstruction group, 41 (50%) patients underwent reconstruction during follow-up. After 24 months, the early ACL reconstruction group had a significantly better (P=0.026) but not clinically relevant International Knee Documentation Committee score (84.7 *v* 79.4 (difference between groups 5.3, 95% confidence interval 0.6 to 9.9). After three months of follow-up, the International Knee Documentation Committee score was significantly better (P=0.002) for the rehabilitation and optional delayed ACL reconstruction group (difference between groups −9.3, −14.6 to −4.0). After nine months of follow-up, the difference in the International Knee Documentation Committee score changed in favour of the early ACL reconstruction group. After 12 months, differences between the groups were smaller. In the early ACL reconstruction group, four re-ruptures and three ruptures of the contralateral ACL occurred during follow-up versus two re-ruptures and one rupture of the contralateral ACL in the rehabilitation and optional delayed ACL reconstruction group.

**Conclusions:**

In patients with acute rupture of the ACL, those who underwent early surgical reconstruction, compared with rehabilitation followed by elective surgical reconstruction, had improved perceptions of symptoms, knee function, and ability to participate in sports at the two year follow-up. This finding was significant (P=0.026) but the clinical importance is unclear. Interpretation of the results of the study should consider that 50% of the patients randomised to the rehabilitation group did not need surgical reconstruction.

**Trial registration:**

Netherlands Trial Register NL 2618.

## Introduction

Rupture of the anterior cruciate ligament (ACL) is a common injury with an acute trauma. This injury leads to a painful swollen knee, with secondary instability complaints, meniscal and chondral damage, and a 10-fold increased risk of osteoarthritis.^1^ ^2^ ^3^ ^4^ ^5^ The incidence is 49-75 per 100 000 person years, with individual and socioeconomic burdens.^6^ ^7^ ^8^ The seminal Knee Anterior Cruciate Ligament, Nonsurgical versus Surgical Treatment (KANON) trial found that non-operative treatment of rupture of the ACL with exercise was successful in at least half of these patients.^9^ ^10^ Early reconstruction of the ACL had a similar functional outcome after two years of follow-up as rehabilitation and optional delayed ACL reconstruction. Ten years on from this seminal publication and clinical practice does not seem to have changed, with the number of ACL reconstructions increasing rather than decreasing.^6^ ^7^ ^8^ Rupture of the ACL must be treated appropriately soon after its traumatic onset, by surgery or by exercise. A good evidence based treatment strategy is important for patients with rupture of the ACL. In contrast with the KANON study, we used the International Knee Documentation Committee score as the primary outcome measure. We previously found that this score had better measurement properties and was therefore more useful than the Knee Injury and Osteoarthritis Outcome questionnaire in evaluating these patients.^11^

The aim of the trial was to assess whether a clinically relevant difference existed in patients’ perceptions of symptoms, knee function, and ability to participate in sporting activities between two commonly used treatment regimens: early reconstruction of the ACL versus rehabilitation and optional delayed ACL reconstruction. The primary outcome was measured with the International Knee Documentation Committee score over a period of two years after rupture of the ACL.

## Methods

### Study design

The Conservative versus Operative Methods for Patients with ACL Rupture Evaluation (COMPARE) trial was an open labelled, multicentre, parallel randomised controlled trial that evaluated the effectiveness of two treatment strategies for acute rupture of the ACL. Patients were recruited between May 2011 and April 2016 at six hospitals (one university hospital and five non-university hospitals) in the Netherlands.

### Patients

Patients were recruited from the outpatient clinic of the Albert Schweitzer Hospital, Erasmus MC University Medical Centre, Haaglanden Medical Centre, Elisabeth Tweesteden Hospital, Reinier de Graaf Gasthuis, and St Antonius Hospital. Patients aged 18-65 with an acute (within two months after the initial trauma) complete primary ACL rupture (confirmed by magnetic resonance imaging and clinical examination) and willing to be randomised were eligible for the trial. Exclusion criteria were a history of injury to the ACL of the contralateral knee, the presence of another disorder affecting the activity of the lower limb, a dislocated bucket handle lesion of the meniscus with an extension deficit, or insufficient command of the Dutch language. Eligible patients received oral and written standardised information about the trial.

### Randomisation and masking

After patients signed the informed consent form and baseline measurements had been carried out, they were randomised to one of two groups. An independent person (central randomisation) had access to the computer generated randomisation lists (block randomisation, with variable sizes of the blocks (range 2-6), stratified by orthopaedic surgeon and age group (<30 and ≥30)).

### Interventions

Patients were randomised to early reconstruction of the ACL or rehabilitation followed by optional delayed reconstruction of the ACL. After randomisation, patients were told of their treatment assignment. The surgeon responsible for the treatment was also informed.

#### Early ACL reconstruction

Arthroscopic reconstruction of the ACL was scheduled within six weeks after randomisation. Surgeons chose their preferred technique and graft, and decided if more intra-articular surgery was necessary. All six participating hospitals had up to two orthopaedic surgeons performing ACL reconstructions; all participating surgeons had a minimum of 10 years’ experience. After surgery, patients were referred for physical therapy until good functional control was achieved.^1^

#### Rehabilitation with optional delayed ACL reconstruction

For non-operative treatment, patients were referred to a physical therapist for a supervised physical therapy programme for a minimum of three months, according to the recommendations of the Dutch ACL guideline.^1^ After a minimum of three months of rehabilitation, patients could opt for reconstruction of the ACL if instability persisted or if the desired activity level was not reached.

### Outcomes

The primary outcome was patients’ perceptions of symptoms, knee function, and ability to participate in sports, measured by the International Knee Documentation Committee score, assessed over a period of 24 months. A higher International Knee Documentation Committee score reflected more favourable ratings for symptoms, knee function, and ability to participate in sporting activities (optimum score 100). The International Knee Documentation Committee score is a valid and responsive tool (that is, it can detect changes over time) for patients with rupture of the ACL.^11^ ^12^ ^13^

Secondary outcomes were: Knee Injury and Osteoarthritis Outcome score (a sum score for each of five subscales for pain, symptoms, activities of daily living, sports, and quality of life; range 0-100, optimum score 100); Lysholm score (range 0-100, optimum score 100); return to sporting level before the injury (yes, no); occurrence of giving way (yes, no); sporting activity level (Tegner score; range 0-10, highest activity score 10); knee pain (numeric rating scale 0-10, optimum score 0); and satisfaction with treatment (five point Likert scale, with satisfied defined as moderate and very satisfied). Serious adverse events (meniscal lesions, complications, and re-interventions) were also secondary outcomes.

Patients were seen at the outpatient clinic at baseline, and at 12 and 24 months. Patients completed a questionnaire at three, six, and nine months after randomisation. All questionnaires were completed digitally and the patient study data were coded by data management software (Gemstracker version 1.6.3, Erasmus MC, Rotterdam, Netherlands).

For the sample size calculation, we used the results of the study of Siebold and colleagues.^14^ Patients with rupture of the ACL waiting to undergo reconstruction of the ACL had a mean preoperative International Knee Documentation Committee Score of 56 (within group standard deviation 13) and a mean score of 90 (standard deviation 10) 19 months after operation. We powered the study to detect a seven point difference between the groups for the International Knee Documentation Committee score (based on an effect size of a minimum of 0.5). With a power of 90% and a type I error rate of 5%, we calculated that we needed 75 patients for each group (150 in total). Taking into account a potential loss to follow-up of 25% over two years, the target sample was 188 patients. Based on a much lower loss to follow-up of less than 10% from the interim report to the grant provider, however, we refined this estimation to 166 patients.

### Statistical analysis

In our primary analysis, patients were analysed according to their randomisation group. To answer our primary research question, we used mixed models to evaluate the difference between the groups in the change in the International Knee Documentation Committee score over the follow-up period, as indicated by the interaction between time point and randomised allocation. The International Knee Documentation Committee score (at baseline and after three, six, nine, 12, and 24 months of follow-up) was used as a dependent variable. The repeated measures and covariance structure was modelled as unstructured. The model was estimated with the restricted maximum likelihood approach. The randomised allocation was used as an independent variable. The follow-up period and the interaction between follow-up and randomised allocation were entered into the model as fixed factors. We adjusted the analysis for potential confounders: sex, body mass index, and age. Randomisation was stratified for orthopaedic surgeon and age group (<30 and ≥30), and these were added as random factors into the model. The model assumptions checked were linearity, homoscedasticity, and normality of residuals. We did not find any violation of the model assumptions.

Secondary analyses included analysis of the difference between the groups in Knee Injury and Osteoarthritis Outcome score, Lysholm score, and pain severity (numeric rating scale at rest and during activity), by mixed models (as described above) at the different time points. Return to sporting level before the injury, satisfaction with treatment, and adverse events were reported as comparative frequencies. Because of the potential for a type I error caused by multiple comparisons, findings for analyses of secondary endpoints should be seen as exploratory. Also, we described (post hoc) the primary outcome groups as early surgical reconstruction of the ACL, non-operative treatment, and delayed surgical reconstruction of the ACL after unsuccessful rehabilitation. Statistical significance was set at the two sided 0.05 level.

### Patient and public involvement

In the absence of an adequate patient association, we formed a panel of patients with rupture of the ACL to review and comment on our study. Our patient panel consisted of three patients with an ACL rupture. The trial set-up was discussed with the patient panel before the subsidy request was submitted. In collaboration with these patients, we templated our study protocol as much as possible to our routine follow-up periods and standard measurements. Since 2010, we have expanded our use of patient participation panels on a regular basis. We plan to disseminate the results of the study to the study participants.

## Results

### Patients

**Fig 1:**
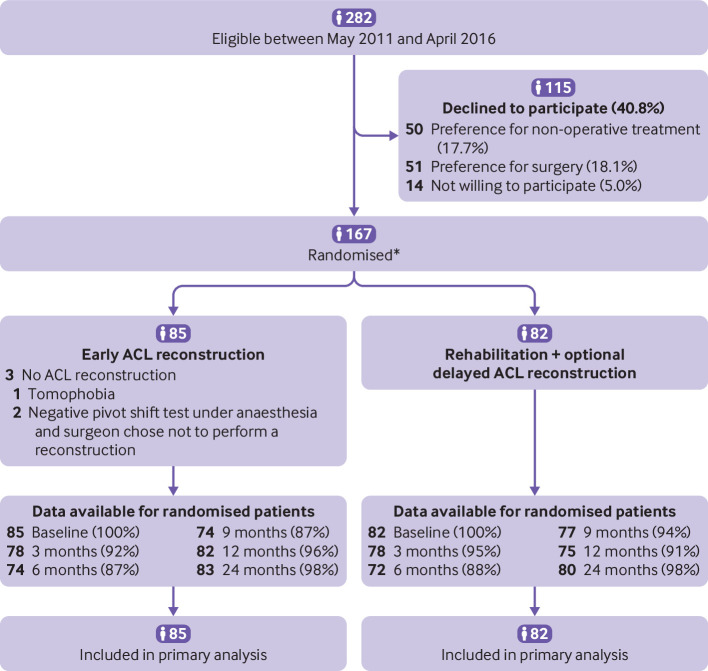
Flowchart of the study participants. *Randomisation by blocks of various sizes by surgeon and age group (<30 and ≥30). ACL=anterior cruciate ligament

Between May 2011 and April 2016, 282 patients were eligible to participate in the study; 115 declined and so 167 patients were enrolled in the study (fig 1). The follow-up period ended in April 2018. Eighty five patients were randomised to the early ACL reconstruction group and 82 to the rehabilitation followed by optional delayed ACL reconstruction group (fig 1, table 1). Sixty one patients had a Tegner score of 9 or 10 before the injury, which represents a high level of sporting activity. Three patients (3.5%) in the early ACL reconstruction group did not undergo reconstruction: one because of tomophobia and two because the surgeon decided not to perform an ACL reconstruction because of a negative pivot shift test during surgery. Of the 82 patients in the early ACL reconstruction group, 78 had an arthroscopic procedure with a hamstring graft and four had a bone patella tendon bone graft.

**Table 1:** Baseline characteristics of the study population

Characteristic	Early ACL reconstruction (n=85)	Rehabilitation+optional delayed ACL reconstruction (n=82)
Age at inclusion	31.2 (10.3)	31.4 (10.7)
No (%) women	36 (42.4)	31 (37.8)
Body mass index	24.3 (3.7)	25.0 (4.1)
No (%) college education	30 (35.3)	36 (43.9)
No (%) paid work	71 (83.5)	64 (78.0)
Tegner score before injury	7.0 (2.3)	7.1 (2.0)
No (%) ACL injured during sport	76 (89.4)	71 (86.6)
Time between injury and inclusion (days; median (IQR))	39.0 (25.5-53.0)	40.5 (29.8-52.5)
No (%) objective anteroposterior knee instability	85 (100)	82 (100)
No (%) MRI findings		
Meniscal tear	38 (44.7)	37 (45.1)
MCL injury	30 (35.3)	31 (37.8)
LCL injury	8 (9.4)	10 (12.2)
Cartilage defect	23 (27.1)	16 (19.5)

Data are mean (standard deviation) unless otherwise stated.

ACL=anterior cruciate ligament; MRI=magnetic resonance imaging; MCL=medial collateral ligament; LCL=lateral collateral ligament; IQR=interquartile range.

Tegner score evaluates sporting activity level (range 0-10, highest activity score 10).

Forty one (50%) patients in the rehabilitation and optional delayed ACL reconstruction group eventually underwent reconstruction during the two years of follow-up, an average of 10.6 months after randomisation. These 41 patients met the criteria for reconstruction of the ACL (that is, occurrence of giving way and rotational instability, confirmed by a positive pivot shift test) recommended by the Dutch guideline.^15^ Of the patients who had delayed reconstruction of the ACL (n=41), 38 had an arthroscopic procedure with a hamstring graft and three patients had a bone patella tendon bone graft. In both groups, no extra-articular tenodesis procedures were performed. Two year follow-up was complete for 98% of all patients.

### Primary outcome

**Fig 2:**
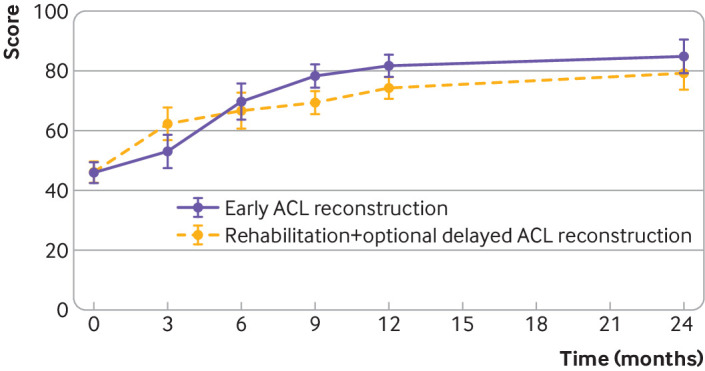
International Knee Documentation Committee Score over a follow-up period of 24 months in the early anterior cruciate ligament (ACL) reconstruction group and the rehabilitation and optional delayed ACL reconstruction group. Values are mean (95% confidence intervals). Data were adjusted for sex, body mass index, age, and surgeon. A significant difference was found in the course of the International Knee Documentation Committee score over the two year follow-up period (P<0.001 for interaction between follow-up and randomised allocation). A higher International Knee Documentation Committee score indicates more favourable patient ratings for symptoms, knee function, and ability to participate in sporting activities (optimum score 100)

Both treatment groups had an improvement in the International Knee Documentation Committee score over the two year follow-up period (fig 2, table 2). We found a significant difference in the course of the International Knee Documentation Committee score over the two year follow-up period (P<0.001 for the interaction between follow-up time and randomised allocation).

**Table 2:** Primary outcome (estimated International Knee Documentation Committee score) for the as randomised analyses for each measurement period*

Primary outcome	Baseline	Follow-up (months)				
		3	6	9	12	24
IKDC score						
Early ACL reconstruction	45.9 (42.4 to 49.4)	53.0 (47.5 to 58.6)	69.6 (63.5 to 75.6)	78.2 (74.3 to 82.2)	81.6 (77.9 to 85.2)	84.7 (78.8 to 90.5)
Rehabilitation+optional ACL reconstruction	46.2 (42.6 to 49.8)	62.3 (56.8 to 67.9)	66.8 (60.9 to 72.8)	69.3 (65.4 to 73.3)	74.4 (70.6 to 78.2)	79.4 (73.6 to 85.2)
Difference between groups	−0.3 (−5.3 to 4.8)	−9.3 (−14.6 to −4.0)	2.8 (−2.9 to 8.5)	8.9 (3.3 to 14.5)	7.1 (1.9 to 12.4)	5.3 (0.6 to 9.9)

Data are mean (95% confidence interval).

ACL=anterior cruciate ligament; IKDC=International Knee Documentation Committee.

Significant difference was found in the course of the IKDC score over the two year follow-up period (P<0.001 for interaction between follow-up and randomised allocation).

A higher International Knee Documentation Committee score indicates more favourable patient ratings for symptoms, knee function, and ability to participate in sporting activities (optimum score 100).

* Adjusted for sex, body mass index, age, and surgeon.

A significant difference (P=0.026) in the International Knee Documentation Committee score at 24 months was found in favour of the early ACL reconstruction group (difference between groups 5.3, 95% confidence interval 0.6 to 9.9). After three months, a significant difference (P=0.002) was found in favour of the rehabilitation and optional delayed ACL reconstruction group (−9.3, −14.6 to −4.0). After nine months of follow-up, the difference in International Knee Documentation Committee score changed in favour of the early ACL reconstruction group (8.9, 3.3 to 14.5). After 12 months of follow-up, the difference between the groups was smaller (7.1, 1.9 to 12.4).

### Secondary outcomes

Patients in the early ACL reconstruction group had a significantly greater Knee Injury and Osteoarthritis Outcome sport score (P=0.039) than the rehabilitation and optional delayed ACL reconstruction group (80.8, 95% confidence interval 75.5 to 86.0 *v* 72.8, 67.4 to 78.2; difference in change score between groups −7.9, 95% confidence interval −15.4 to −0.4) and a significantly better quality of life score (76.6, 71.8 to 81.4 *v* 65.8, 60.8 to 70.7; difference in change score between groups −10.9, −17.2 to −4.0, P=0.002) at the two year follow-up (table 3). The Knee Injury and Osteoarthritis Outcome scores for the other subscales were not significantly different between the two groups.

**Table 3:** Secondary outcomes at the two year follow-up

Secondary outcome	Early ACL reconstruction (n=83)			Rehabilitation+optional delayed ACL reconstruction (n=80)		Difference between groups in change scores
	Baseline	Two year follow-up		Baseline	Two year follow-up	
KOOS						
Pain	59.8 (52.8 to 66.8)	90.5 (83.5 to 97.5)		60.5 (53.5 to 67.5)	87.1 (80.2 to 94.0)	3.4 (−0.7 to 7.5)
Symptoms	55.8 (49.3 to 62.4)	86.8 (80.4 to 93.2)		49.9 (43.3 to 56.5)	82.5 (76.2 to 88.8)	4.3 (−0.5 to 9.1)
Activities of daily living	65.2 (57.4 to 72.9)	93.6 (85.8 to 101.5)		66.6 (58.8 to 74.3)	92.0 (84.2 to 99.8)	1.6 (−1.3 to 4.6)
Sport	27.5 (22.1 to 33.0)	80.8 (75.5 to 86.0)*		29.2 (23.6 to 34.8)	72.8 (67.4 to 78.2)*	−7.9 (−15.4 to −0.4)†
Quality of life	30.4 (26.5 to 34.2)	76.6 (71.8 to 81.4)*		30.9 (27.0 to 34.9)	65.8 (60.8 to 70.7)*	−10.9 (−17.2; −4.0)†
No (%) with occurrence of giving way	—	2/81 (2.5)*		—	12/80 (15.0)*	—
No (%) with return to sporting level before injury	—	35/81 (43.2)		—	25/80 (31.3)	—
No (%) satisfied with treatment	—	75/81 (92.6)		—	73/80 (91.3)	—

Data are mean (95% confidence interval) unless otherwise stated.

KOOS=Knee Injury and Osteoarthritis Outcome score (range 0-100; optimum score 100).

* Comparison of the two year follow-up values between the groups, P<0.05.

† P<0.05.

For the Lysholm score, we found a significantly higher score after six (P=0.019), nine (P=0.040), 12 (P=0.030), and 24 months (P=0.041) of follow-up in the early ACL reconstruction group (table 4). Pain severity at rest and during activity were not significant different between the groups at any time point.

**Table 4:** Secondary outcomes for the as randomised analyses for each measurement period

Secondary outcome	Baseline	Follow-up (months)				
		3	6	9	12	24
Lysholm score						
Early ACL reconstruction	64.5 (60.5 to 68.6)	77.3 (71.8 to 82.8)	86.6 (79.7 to 93.5)	88.8 (84.9 to 92.8)	90.3 (87.8 to 92.9)	90.6 (85.4 to 95.9)
Rehabilitation+optional ACL reconstruction	64.7 (60.6 to 68.9)	79.6 (74.1 to 85.1)	81.4 (78.9 to 85.9)	84.6 (80.7 to 88.5)	86.2 (83.5 to 88.9)	87.1 (81.9 to 92.3)
Difference between groups	−0.2 (−6.0 to 5.6)	−2.3 (−6.8 to 2.2)	5.2 (0.9 to 9.5)	4.2 (0.2 to 8.2)	4.1 (0.4 to 7.8)	3.5 (0.2 to 6.9)
NRS at rest						
Early ACL reconstruction	2.5 (1.7 to 3.3)	1.4 (0.8 to 2.0)	0.9 (0.4 to 1.4)	0.9 (0.4 to 1.4)	1.0 (0.5 to 1.4)	0.5 (0.1 to 1.0)
Rehabilitation+optional ACL reconstruction	2.3 (1.5 to 3.1)	1.4 (0.7 to 2.0)	1.2 (0.7 to 1.7)	1.4 (0.9 to 1.9)	1.3 (0.8 to 1.7)	0.9 (0.4 to 1.3)
Difference between groups	0.2 (−0.5 to 0.9)	0 (−0.6 to 0.7)	−0.3 (−0.8 to 0.2)	−0.4 (−1.0 to 0.1)	−0.3 (−0.9 to 0.3)	−0.3 (−0.8 to 0.1)
NRS during activity						
Early ACL reconstruction	5.0 (4.4 to 5.6)	3.6 (2.6 to 4.6)	2.7 (2.0 to 3.4)	2.4 (1.8 to 3.1)	1.8 (1.2 to 2.4)	1.6 (1.0 to 2.3)
Rehabilitation+optional ACL reconstruction	5.1 (4.5 to 5.7)	3.9 (3.0 to 4.9)	3.0 (2.3 to 3.7)	2.9 (2.2 to 3.5)	2.7 (2.0 to 3.3)	2.2 (1.6 to 2.9)
Difference between groups	−0.2 (−1.0 to 0.7)	−0.3 (−1.1 to 0.4)	−0.3 (−1.1 to 0.4)	−0.4 (−1.1 to 0.3)	−0.9 (−1.6 to −0.2)	−0.6 (−1.2 to 0)

Data are mean (95% confidence interval).

ACL=anterior cruciate ligament; NRS=numeric rating scale for knee pain (0-10, optimum score 0).

Lysholm score (range 0-100; optimum score 100).

### Other treatments

In the early ACL reconstruction group, 24 arthroscopic meniscus procedures (18 meniscectomies, four repairs, and two both procedures) were performed during reconstruction of the ACL compared with 17 in the rehabilitation and optional delayed ACL reconstruction group (11 meniscectomies, five repairs, and one both procedures). One meniscectomy procedure in the early ACL reconstruction group was performed before the ACL reconstruction session.

### Serious adverse events

Table 5 shows the number of serious adverse events for the two treatment groups. In the early ACL reconstruction group, three ruptures of the contralateral ACL occurred compared with one in the rehabilitation and optional delayed ACL reconstruction group. Four re-ruptures occurred in the early ACL reconstruction group and two in the rehabilitation and optional delayed ACL reconstruction group.

**Table 5:** Serious adverse events

Adverse event	Early ACL reconstruction (n=85)	Rehabilitation+optional delayed ACL reconstruction (n=82)
Re-rupture of ACL reconstruction (No)	4	2
Rupture of contralateral ACL (No)	3	1
Removal of tibial screw (No)	1	2
Arthroscopic intervention for meniscal tear after ACL reconstruction or new knee trauma (No)	4	3
Arthroscopic debridement for extension deficit (No)	2	4

ACL=anterior cruciate ligament.

### Post hoc analysis

The post hoc as treated evaluations of the recovery of the International Knee Documentation Committee score for the three groups of patients are reported in eFigure 1 and eTable 1 (supplements 1 and 2). Giving way complaints were present substantially more often in the rehabilitation and optional delayed ACL reconstruction group than in the early ACL reconstruction group after two years of follow-up (15.0% *v* 2.5%, respectively).

## Discussion

### Principal findings

In this multicentre, randomised controlled trial of treatment of acute injury to the ACL, we found that patient who underwent early surgical reconstruction, compared with rehabilitation followed by optional surgical reconstruction, had improved perceptions of symptoms, knee function, and ability to participate in sports after two years of follow-up. The effect was significant (P=0.026) but the clinical importance is unclear. Interpretation of the results of the study should consider that 50% of the patients randomised to the rehabilitation group did not need surgical reconstruction.

### Comparison with other studies

Half of the patients in our study required surgical reconstruction of the ACL after unsuccessful rehabilitation. The KANON trial showed that after two years of follow-up, 39% of patients had reconstruction of the ACL, which increased to 51% after five years of follow-up.^9^ ^10^ The study population of the KANON trial, however, was younger (about five years) and had a higher sporting level (9 *v* 7) before the injury. Publication of the results of the KANON trial does not seem to have affected the decision to operate because the number of ACL reconstructions is still increasing worldwide. But our results and the results of the KANON trial showed that reconstruction is not necessary in at least half of the patients. In daily practice, another reason to choose surgical reconstruction is that recurrent giving way episodes can lead to secondary injuries of the meniscus and cartilage. We found more surgical interventions for a meniscal tear in the early ACL reconstruction group. Also, after reconstruction of the ACL, meniscus procedures were performed because of a new trauma, suggesting that surgical reconstruction will not decrease this risk.

Patients with rupture of the ACL have an increased risk of knee osteoarthritis.^16^ Which treatment option is best for preventing the development of osteoarthritis is still unclear. The evidence is conflicting, as reported by our group in 2015 and in other studies.^16^ ^17^ Longer follow-up of our study is needed to evaluate the long term risk of knee osteoarthritis.

A difference of seven points between the two groups in the International Knee Documentation Committee score was used to assess the number of patients needed in our study. This number was based on an effect size of 0.5, which is described as a medium effect. During the preparation of our study, information on the minimal clinically important difference in the International Knee Documentation Committee score was not available. Since then, several papers have reported minimal clinically important differences of 11.5-20.5 in patients who have undergone surgical procedures for various knee pathologies.^18^ ^19^ In our study, differences between the groups at any time point did not exceed the lowest reported minimal clinically important difference. Also, after two years of follow-up, differences between the groups did not exceed the lowest reported minimal clinically important difference or our predefined difference, implying that the clinical relevance of this difference is uncertain.

Because 50% of the patients in the rehabilitation group opted for delayed reconstruction implies that these patients were not satisfied with the results of conservative treatment. The next question is whether these patients would have benefitted from early reconstruction. Therefore, future research should be directed towards timely and correct identification of these patients in the acute stage, and this group of patients undergoing early reconstruction with rehabilitation and patients undergoing optional delayed reconstruction should be compared prospectively. This research is challenging because of some of the views about the treatment of patients with rupture of the ACL (eg, that patients with a high level of activity always require reconstruction).^20^

The lower International Knee Documentation Committee scores during follow-up in the delayed ACL reconstruction group suggest that the best treatment for a patient with rupture of the ACL is early reconstruction of the ACL or rehabilitation with physiotherapy. The findings in eFigure 1 (supplement 2) should be interpreted with caution, however.

The group that had delayed surgery was a selected subgroup, and compared with the group that had early surgery or with the group that did not opt for delayed surgery is likely biased and should not be formally tested. eFigure 1, however, shows the course of these three groups; half of the patient in the rehabilitation group did well over the two year period whereas the other half that opted for delayed surgery did less well before surgery and recovered only slowly after surgery. Also, many patients in the delayed ACL reconstruction group underwent surgery in the second year and consequently were still in the rehabilitation stage when evaluated at the two year follow-up.

We found three ruptures of the contralateral ACL in the early ACL reconstruction group and one in the rehabilitation and optional ACL reconstruction group during the two years of follow-up (table 5). A possible explanation is that participants in the early ACL reconstruction group had finished rehabilitation after the surgical procedure (about 12 months) and had returned to sporting activities. In contrast, participants in the optional ACL reconstruction group were still in the rehabilitation stage and had not returned to sporting activities.

The reported ruptures of the contralateral ACL are similar to those in other studies. A study of national hospital data for all reconstructions of the ACL performed in England between 1 April 1997 and 31 March 2017 found that 2.9% (95% confidence interval 2.7 to 3.0) underwent ACL reconstruction of the contralateral knee.^21^

### Strengths

Our study had several strengths. Firstly, we included a large number of patients willing to participate in a study in which they were randomised to surgery or non-operative treatment. Recruiting patients willing to participate in a randomised controlled trial, particularly when a surgical intervention is compared with a non-operative intervention, is challenging.^22^ Because of the difficulty of including patients in this type of study, we believe that our study will not be repeated in the near future. Secondly, we used a different primary outcome measure than the KANON study. We previously found that the International Knee Documentation Committee score had better measurement properties and was therefore more useful than the Knee Injury and Osteoarthritis Outcome questionnaire in evaluating these patients.^11^ Thirdly, the high follow-up rate and few protocol violations strengthen the validity of our outcomes. Fourthly, the multicentre design of our study implies that the results of the study are applicable to many patients.

### Limitations

Our study had some limitations. Firstly, recruitment bias could be present because of the 282 eligible patients, 101 declined to participate in the study because of a strong preference for one of the treatment options (51 preferred surgery and 50 preferred non-operative treatment). Because these preferences were equally divided, the results of our study would likely not have been different if all eligible patients had participated. The group that had delayed surgery was a selected subgroup, and comparisons with the group that had early surgery or with the group that did not opt for delayed surgery were probably biased and consequently were not formally tested. 

### Conclusions

In patients with acute rupture of the ACL, those who underwent surgical reconstruction alone, compared with rehabilitation and optional surgical reconstruction, had improved perceptions of symptoms, knee function, and ability to participate in sports at the two year follow-up. This finding was significant (P=0.026) but the clinical importance is unclear. Interpretation of the results of the study should consider that 50% of the patients randomised to the rehabilitation group did not need surgical reconstruction.

**What is already known on this topic**

Several non-randomised studies have suggested that for patients with rupture of the anterior cruciate ligament (ACL), clinical results for early reconstruction of the ACL compared with rehabilitation alone are similar
Evidence from randomised controlled trials is lacking
The preferred treatment for rupture of the ACL (surgery or rehabilitation) is unclear

**What this study adds**

Patients who underwent early surgical reconstruction of the ACL, compared with those who had rehabilitation followed by elective surgical reconstruction, had improved perceptions of symptoms, knee function, and ability to participate in sports at the two year follow-up
This finding was significant but the clinical importance is unclear
Interpretation of the results of the study should consider that 50% of patients randomised to the rehabilitation group did not need surgical reconstruction

## Web extra

material supplied by authors

Web appendix 1: Supplement 1

Web appendix 2: Supplement 2

## Notes

Contributors: MR, VE, and DM conceived and designed the study. All authors acquired, analysed, or interpreted the data. EW and MR performed the statistical analysis. MR, VE, and DM drafted the manuscript. All authors critically revised the manuscript for important intellectual content. MR and DM are the guarantors. MR is the corresponding author and attests that all listed authors meet authorship criteria and that no others meeting the criteria have been omitted.

Ethical approval: This study was approved by the medical ethics committee of the Erasmus MC, Rotterdam, Netherlands (MEC-2010-291). All patients gave written informed consent.

Data sharing: Individual deidentified participant data that underlie the results reported in this paper (text, tables, figures, and appendices) and the study protocol will be shared if requested. Data will be available beginning 12 months and ending five years after publication of this paper. Data will be available for researchers who provide a methodologically sound scientific proposal, which has been approved by an ethical committee. Proof of the latter should be provided. Analyses should achieve the aims reported in the approved proposal. Proposals for data should be directed to the corresponding author (m.reijman@erasmusmc.nl).

The lead author (MR) affirms that the manuscript is an honest, accurate, and transparent account of the study being reported; that no important aspects of the study have been omitted; and that any discrepancies from the study as planned (and, if relevant, registered) have been explained.

Dissemination to participants and related public communities: Recruited patients have been informed that the results of the trial will not be sent to individuals but will be available after publication on request to the corresponding author (MR).

Provenance and peer review: Not commissioned; externally peer reviewed.
