# Supplementary material for: Early surgical reconstruction versus rehabilitation with elective delayed reconstruction for patients with anterior cruciate ligament rupture: COMPARE randomised controlled trial
Source: BMJ. 2021 Mar 9;372:n375. doi: 10.1136/bmj.n375 (PMC7941216; doi:10.1136/bmj.n375)
Supplement: Supplementary file 1 — Web appendix 1: Supplement 1 [file reim061484.ww1.pdf]

eTable 1: post-hoc as-treated results for primary outcome (estimated International Knee Documentation Committee Score (**IKDC[a]))**

	**baseline**	**FU 3 months**	**FU 6 months**	**FU 9 months**	**FU 12 months**	**FU 24 months**
**IKDC**						
- Early ACL reconstruction	47.1 (43.5; 50.7)	53.4 (50.1; 56.8)	69.0 (65.5; 72.6)	77.4 (73.7; 81.1)	81.2 (77.9; 84.5)	85.9 (82.6; 89.1)
- Delayed ACL reconstruction	50.7 (45.3; 56.0)	58.5 (53.8; 63.2)	59.2 (54.3; 64.1)	61.1 (56.0; 66.2)	67.1 (62.4; 71.9)	78.4 (73.7; 83.1)
- Non-operative	43.3 (37.9; 48.8)	69.1 (64.3; 73.8)	76.4 (71.3; 81.5)	77.9 (72.7; 83.1)	82.5 (77.6; 87.5)	83.5 (78.8; 88.3)

Data are presented as mean and 95% confidence interval between parentheses

a adjusted for sex, BMI, and age

FU = follow-up

A higher International Knee Documentation Committee score reflects more favourable patients ratings of symptoms knee function and ability to participate in sports activities (optimal score is 100)
